# Manual Thrombus Aspiration and the Improved Survival of Patients With Unstable Angina Pectoris Treated With Percutaneous Coronary Intervention (30 Months Follow-Up)

**DOI:** 10.1097/MD.0000000000002919

**Published:** 2016-03-03

**Authors:** Bekir S. Yildiz, Murat Bilgin, Mustafa Zungur, Yusuf I. Alihanoglu, Ismail D. Kilic, Ipek Buber, Ahmet Ergin, Havane A. Kaftan, Harun Evrengul

**Affiliations:** From the Pamukkale University Medical Faculty, Department of Cardiology (BSY, YIA, IDK, IB, HAK, HE), Denizli; Dıskapı Training and Research Hospital, Department of Cardiology (MB), Ankara; Sifa University Medical Faculty, Department of Cardiology (MZ), Izmir; and Pamukkale University Medical Faculty, Department of Public Health (AE), Denizli, Turkey.

## Abstract

The clinical effect of intracoronary thrombus aspiration during percutaneous coronary intervention in patients with unstable angina pectoris is unknown. In this study, we aimed to assess how thrombus aspiration during percutaneous coronary intervention affects in-hospital and 30-month mortality and complications in patients with unstable angina pectoris.

We undertook an observational cohort study of 645 consecutive unstable angina pectoris patients who had performed percutaneous coronary intervention from February 2011 to March 2013. Before intervention, 159 patients who had culprit lesion with thrombus were randomly assigned to group 1 (thrombus aspiration group) and group 2 (stand-alone percutaneous coronary intervention group). All patients were followed-up 30 months until August 2015.

Thrombus aspiration was performed in 64 patients (46%) whose cardiac markers (ie, creatinine kinase [CK-MB] mass and troponin T) were significantly lower after percutaneous coronary intervention than in those of group 2 (CK-MB mass: 3.80 ± 1.11 vs 4.23 ± 0.89, *P* = 0.012; troponin T: 0.012 ± 0.014 vs 0.018 ± 0.008, *P* = 0.002). Left ventricular ejection fraction at 6, 12, and 24 months postintervention was significantly higher in the group 1. During a mean follow-up period of 28.87 ± 6.28 months, mortality rates were 6.3% in the group 1 versus 12.9% in the group 2. Thrombus aspiration was also associated with significantly less long-term mortality in unstable angina pectoris patients (adjusted HR: 4.61, 95% CI: 1.16–18.21, *P* = 0.029).

Thrombus aspiration in the context of unstable angina pectoris is associated with a limited elevation in cardiac enzymes during intervention that minimises microembolization and significantly improves both of epicardial flow and myocardial perfusion, as shown by angiographic TIMI flow grade and frame count. Thrombus aspiration during percutaneous coronary intervention in unstable angina pectoris patients has better survival over a 30-month follow-up period.

## INTRODUCTION

Unstable angina pectoris (UAP) is the subset of acute coronary syndromes (ACS) caused by the erosion or rupture of atherosclerotic plaque and thrombosis.^1,2^ The prevalence of coronary thrombus has been estimated at 10% to 80% in patients with UAP.^3^ Primary therapy is often urgently needed and/or involves elective percutaneous coronary intervention (PCI).^2^ During PCI, the percutaneous dilatation of coronary stenosis inevitably causes plaque disruption, which may in turn cause the distal embolization of plaque debris or thrombus material. Myocardial tissue reperfusion often reduces due to distal vascular debris embolization that prompts the plugging of the microvasculature, microvascular dysfunction, and myocardial necrosis.^4^

Recent studies using thrombectomy or a distal protection apparatus in primary PCI for ST segment elevation myocardial infarction (STEMI) have demonstrated that using such apparatuses can significantly reduce the incidence of distal embolization and improve both myocardial perfusion and clinical outcomes.^5–9^ Favorable outcomes have also been reported in patients with non-STEMI (NSTEMI) if angiography suggested the presence of thrombus formation.^10^ That study's analysis included PCI patients with UAP, in each of whom the application of manual aspiration catheter for thrombus aspiration (TA) during PCI was insufficient.^11^ Currently, no published data comparing TA in patients with UAP are available. The primary aim of this observational study was to assess how TA during PCI effects in-hospital and 30-month mortality in UAP patients. Its secondary aim was to assess this effect in relation to regional and global contractile left ventricular (LV) function, as well as examine how TA impacts on post-PCI thrombolysis in terms of myocardial infarction (TIMI) flow, TIMI frame count (TFC), and myocardial blush grade (MBG).

## MATERIALS AND METHODS

### Study Design and Population

The present research entailed a multicenter, retrospective, observational, cohort study involving the blind evaluation of end points confirmed by the local ethics committee and in accordance with the Declaration of Helsinki. We retrospectively studied 159 patients selected among 645 UAP patients consecutively referred to the catheterization laboratory of our institutions for coronary angiography and angioplasty. The analysis comprised PCI patients with UAP in each of whom the export aspiration catheter was applied for TA during PCI at Pamukkale University and Sifa University between February 15, 2011 and March 1, 2013. All patients were followed-up 30 months until August 1, 2015 except death.

**FIGURE 1:**
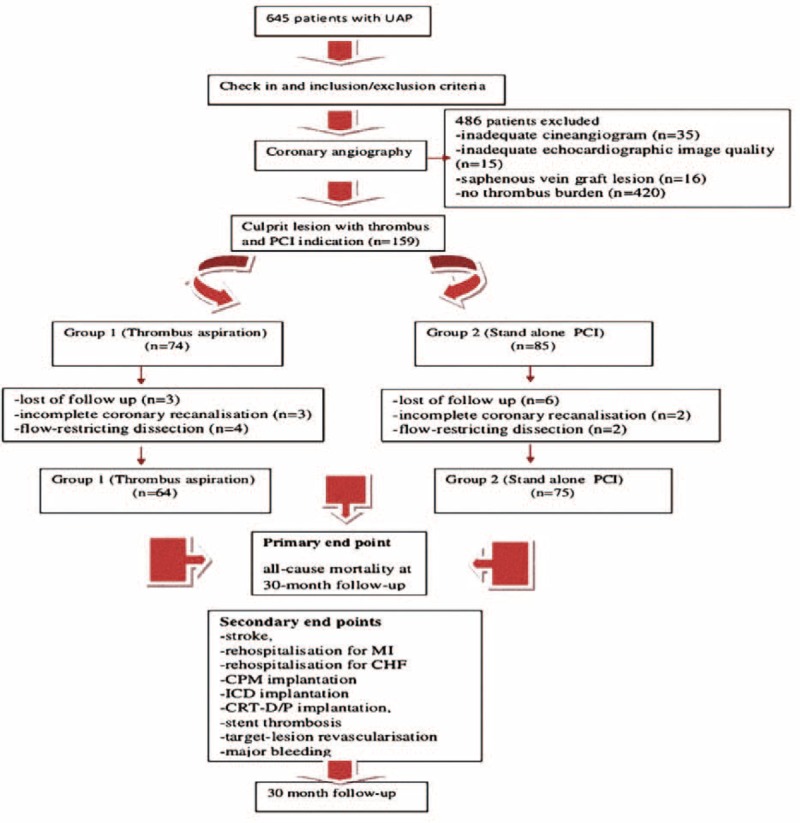
Flow diagram of the study profile.

UAP was defined as the occurrence of typical chest pain at rest with or without electrocardiographic signs of ischemia yet without ST elevation or increase in cardiac troponin T greater than 0.01 ng/ml and creatinine kinase-MB (CK-MB) mass greater than 5 ng/ml (ie, local laboratory threshold of myocardial infarction). Inclusion criteria for UAP patients were thrombus burden vessel diameter greater than 2.5 mm, and technical viability for angioplasty independent of both initial TIMI flow and angiographic evidence of intraluminal thrombus in the culprit artery. Thrombus was defined as the presence of a roundish filling defect of the lumen during dye injection (in multiple projections either) with or without the persistence of luminal contrast following injection. Patients who had been taking anticoagulants and presented with cardiogenic shock and/or thrombus formation as a complication of a PCI (eg, vessel closure after stenting) were excluded from the sample. Other exclusion criteria were the known existence of a disease resulting in a life expectancy less than 6 months, and the lost of patient during follow-up. Patients with echocardiographic image of a poor quality were not accepted for the study. No other clinical exclusion criteria were adopted. The echocardiographic acoustic window was assessed in the emergency department or catheter laboratory by a staff cardiologist before the procedures. After enrollment and before PCI, 159 patients who had culprit lesion with thrombus were randomly assigned to group 1 (TA group) and group 2 (stand-alone PCI group) according to a computer-generated random series of numbers (Figure 1).

### Procedure

In all patients catheterization was performed following the femoral approach by experienced cardiologists. To begin, a steerable guide wire was passed through the target lesion; direct stenting was left to the operator's discretion and usually performed according to patent vessel with no or mild calcification. In patients in the group 2, this step was followed by balloon dilation to assure antegrade flow where as in those in the group 1, the step was followed by advancing the 6F export aspiration catheter (Medtronic, Minneapolis, MN), a 6F-compatible TA catheter with an aspiration lumen of 0.041 in. and cross profile of 0.068 in., which was handled over a guide wire of 0.014 in. in a monorail fashion. Suction was performed manually with lockable 20-ml syringes. After crossing the lesion with a guide wire, the export aspiration catheter was advanced into the target segment during continuous aspiration. The number of passages necessary to achieve an optimal result was left to the judgment of the operator, but at least 2 × 20 ml was aspirated in multiple passages. Additional balloon angioplasty was performed when necessary for stent delivery. After restoring the antegrade flow, in patients deemed suitable intracoronary nitrates were given to achieve maximal epicardial vasodilation, largely to determine the length, and size of the stent and facilitate stent placement.^5^ After placement, PCI postprocedural peak creatinine kinase MB mass (CK-MB) mass and troponin T level were measured. Measurement of CK-MB mass and troponin T were also repeated in 6, 24, and 48 h after PCI. Totally cardiac enzymes were measured 4 times after PCI.

### Medication

Pharmacological treatment before and after PCI was performed according to European Society of Cardiology guideline.^12^

### Angiographic and Echocardiographic Analysis

Coronary angiograms were performed digitally (INNOVA 2100, GE Healthcare, Waukesha, WI). Calibration of the quantitative coronary angiography (QCA) system was carried out by method in which the coronary catheter employed for angiography and was used as the calibration object by automated edge detection technique resulting in corresponding calibration factors (mm/pixel). The coronary artery contour was detected by operator independent edge detection algorithms. The dimension of the coronary artery was then measured as a function of catheter diameter; the absolute diameter in mm was calculated by the computerized software analysis. At least 2 orthogonal projections of the coronary segment scheduled for coronary intervention were filmed before and after the intervention. All angiograms were evaluated and reviewed offline by 2 experienced, interventional cardiologists blind both the results and success of the technique, as stated in the procedural report, for the presence of intraluminal filling defects and TIMI flow grade before PCI, after TA, and at the end of the procedure. The feasibility, success rate, and occurrence of potential complications (eg, coronary dissection and perforation) due to TA were reported. The applicability of the aspiration procedure was defined as the ability to advance the aspiration catheter into the target lesion without predilatation, while the accomplishment of TA was defined as the visually determined reduction of intraluminal filling defects in the angiogram and/or the presence of visible thrombus in the aspirate. Improvement in coronary flow due to TA was defined as the improvement in TIMI flow grade by at least one grade. Distal embolization during PCI was defined as the presence of a new distal occlusion of either the treated vessel or one of its side branches or the migration of a filling defect. No reflow was defined as TIMI flow grade 0 to 1 not due to occlusive thrombus formation, dissection, or coronary artery spasm. Baseline, postaspiration, and final post-PCI angiographic coronary flows were also assessed by means of the corrected TFC at 12.5 frames/s.^13^ Baseline and final post-PCI MBGs were determined.^14^ Clinical status in terms of stroke, reinfarction, bleeding, pacemaker implantation, rehospitalization for heart failure (HF), target vessel revascularization, and death was determined from hospital records as well as by face to face interviews at 1, 6, 12, 24, and 30 months after the procedure.

LV ejection fraction (LVEF) and LV volumes were measured with the modified Simpson rule algorithm by echocardiography.^15^ The mean value of 3 measurements of the technically best cardiac cycles was taken from each examination. LVEF and LV volume changes at 6, 12, and 24 months were compared within 24 h of admission. Intraobserver and interobserver variability values in the evaluation of end-diastolic and -systolic volumes were <5%, which suggests the good reproducibility of the measurements.^16^

### Hospital Complications and Study End Points

In-hospital complications such as recurrent MI, stent thrombosis, atrial fibrillation, ventricular fibrillation, major bleeding (hemoglobin drop of 3–5 g% or need for blood transfusion, a drop in hemoglobin ≥5 g%, cardiac tamponade, need for surgical treatment or hemodynamic instability, and intracranial or intraocular bleeding), stroke, and acute renal failure were noted, in addition to 30-month mortality, complications, and major adverse cardiovascular events over a 30-month follow-up in patients undergoing PCI. The 30-month follow-up was 95% complete.

The primary end point was all-cause mortality at 30-month follow-up. We here report the following prespecified secondary end points: stroke, rehospitalization for MI and congestive heart failure (CHF), cardiac pacemaker (CPM), implantable cardioverter defibrillator (ICD) or cardiac resynchronization therapy and defibrillator/pacemaker (CRT-D/P) implantation, stent thrombosis, and target-lesion revascularization. The incidence of bleeding complications was assessed both during PCI and at 30 months following PCI.

### Statistical Analysis

For quantitative variables, M and SD were calculated. Discrete variables are here presented as the number of events and their percentages. Comparisons were made with Chi-square or Fisher exact tests for discrete variables and by unpaired *t* tests, Wilcoxon sign-rank tests, or 1-way analyses of variance (ANOVA) for continuous variables. Repeated measured ANOVA was used for repeatedly measured variables. Survival curves were estimated using the Kaplan–Meier estimator and compared using log-rank tests while correlates of 30-month survival were determined using a multivariate backward stepwise Cox analysis. Cumulative hazard functions were computed to assess proportionality, and differences were considered significant at *P* < 0.05. Statistical analyses were performed by using the Statistical Package for the Social Sciences for Windows version 17 (SPSS, Chicago, IL).

## RESULTS

### Patient and Procedural Data

A total of 139 UAP patients (mean age 65.02 ± 13.00 years, 69.1% male) were studied, 64 of whom 46% underwent thrombectomy. TA was attempted before PCI in UAP patients (aged 64.28 ± 12.7 years) who had angiography-detected of thrombus formation. Four hundred eighty-six patients were excluded from analysis (Figure 1). There were no statistically significant between-group differences in age, sex, duration of chest pain, risk factors (ie, body mass index, diabetes mellitus, hypertension, hyperlipidemia, smoking, renal insufficiency, family history and history of coronary artery disease [CAD], coronary artery bypass grafting [CABG], cerebrovascular accident, CHF, and peripheral artery disease), ECG parameters (ie, heart rate and rhythm at admission), blood parameters, drugs or medication taken before admission, LVEF within 24 h, Global Registry of Acute Coronary Events (GRACE) score, and Killip classification at admission. Baseline demographics and clinical characteristics are shown in Table 1.

**TABLE 1:**
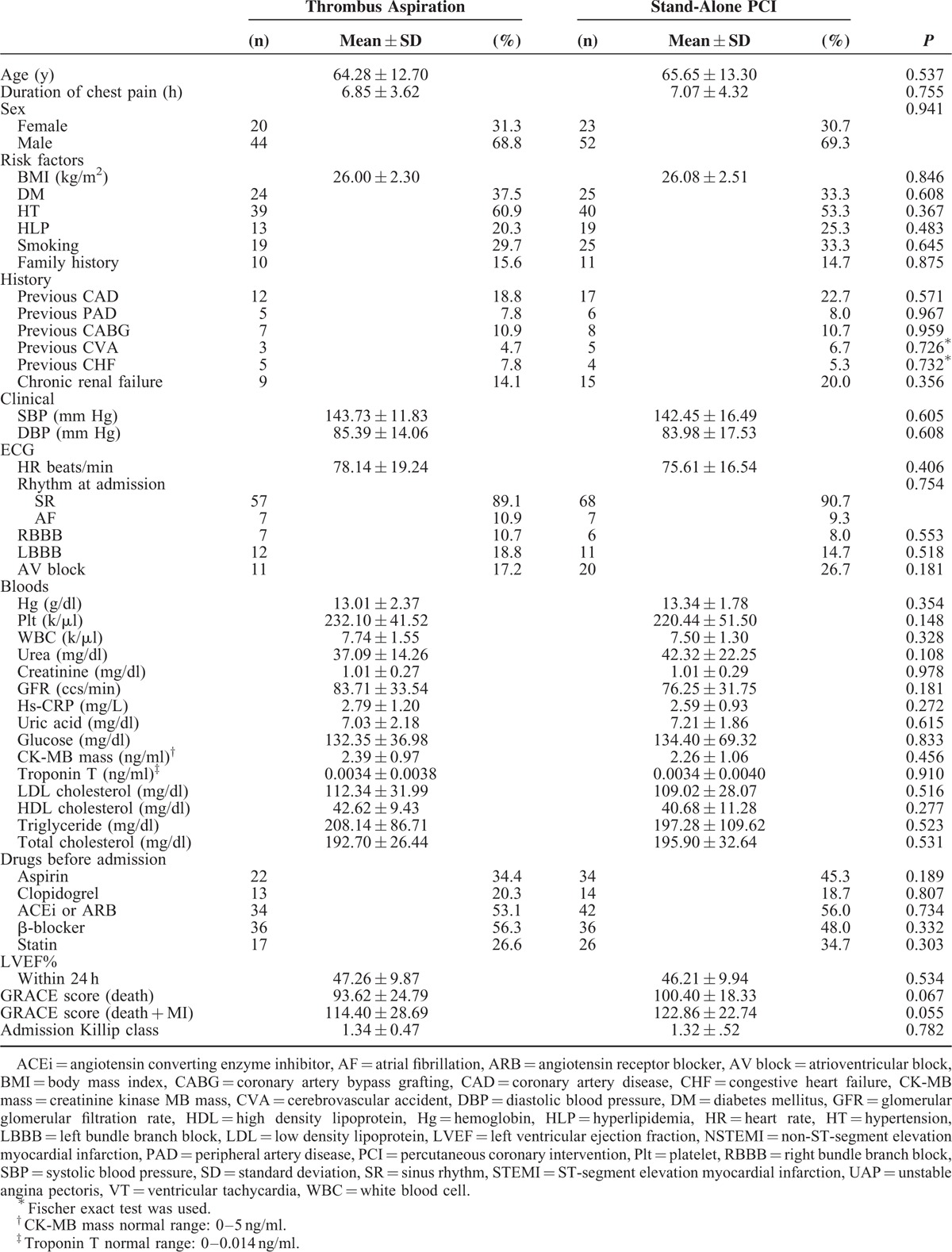
Baseline Characteristics of Study Population

The culprit coronary artery was the right coronary artery (RCA) in 34.4% of the group 1 and in 22.7% of the group 2, the left anterior descending artery (LAD) in 48.4% and 56% of the 2 groups, the left circumflex artery (Cx) in 15.6% and 18.7%, and the left main coronary artery (LMCA) in 1.6% and 2.7% (*P* < 0.486). Three and more vessel disease was present in 17.2% of patients in the group 1 versus 28% of patients in the group 2. Stent implantation was performed in 92.2% of patients in the group 1 and in 98.7% in the group 2. The use of a drug-eluting stent (DES) was similar in both groups (*P* = 0.268). Direct stenting was performed in 46 patients (34%). Cardiac markers (ie, CK-MB mass and troponin T) were significantly lower after PCI in the group 1 than the group 2 (CK-MB mass: 3.80 ± 1.11 vs 4.23 ± 0.89, *P* = 0.012; troponin T: 0.012 ± 0.014 vs 0.018 ± 0.008, *P* = 0.002). There were no statistically significant between-group differences in occlusion pre-PCI, balloon angioplasty, balloon length and diameter, balloon inflation time, indeflator pressure, stent diameter and length, stent balloon inflation time, indeflator pressure for stent balloon, stent postdilatation, or procedure complication (eg, coronary dissection, coronary perforation, hematoma, and arteriovenous fistula). Duration of hospitalization was statistically briefer in the group 1 than in the group 2 (4.25 ± 4.02 day vs 6.49 ± 5.83 day, *P* = 0.011). The use of anticoagulants and antiaggregants was generally similar between both groups, as was treatment by statins, β-blockers, ACEi, or ARB during the first 24 h. TA was associated with an increased rate of TIMI-flow 3 (37.5% in group 1 and 34.7% in the group 2 preprocedure vs 93.8% and 78.7%, respectively, at the end of procedure). The number of patients with postoperative TIMI grade 3 blood flow was significantly higher in the group 1 (*P* = 0.036). The number of patients with a no-reflow rate was lower in the group 1 than in the group 2, though not significantly (*P* = 0.687) while the incidence of MBG 3 was 90.6% and 70.3%, respectively (*P* = 0.031). TFCs were significantly decreased in infarct related arteries in both groups after PCI. The decrease in TFCs was far greater in the group 1 than in the group 2 and statistically significant (Table 2 ). Furthermore, a significant decrease in TFCs also emerged following aspiration in all coronary arteries in group 1 (TFC-LAD preaspiration: 25.93 ± 4.46 vs postaspiration: 17.25 ± 2.17, *P* = 0.001; TFC-CX preaspiration: 20.07 ± 2.46 vs postaspiration: 14.35 ± 2.06, *P* = 0.001; TFC-RCA preaspiration: 18.20 ± 5.33 vs postaspiration: 14.65 ± 2.59, *P* = 0.001) (Table 3).

**TABLE 2:**
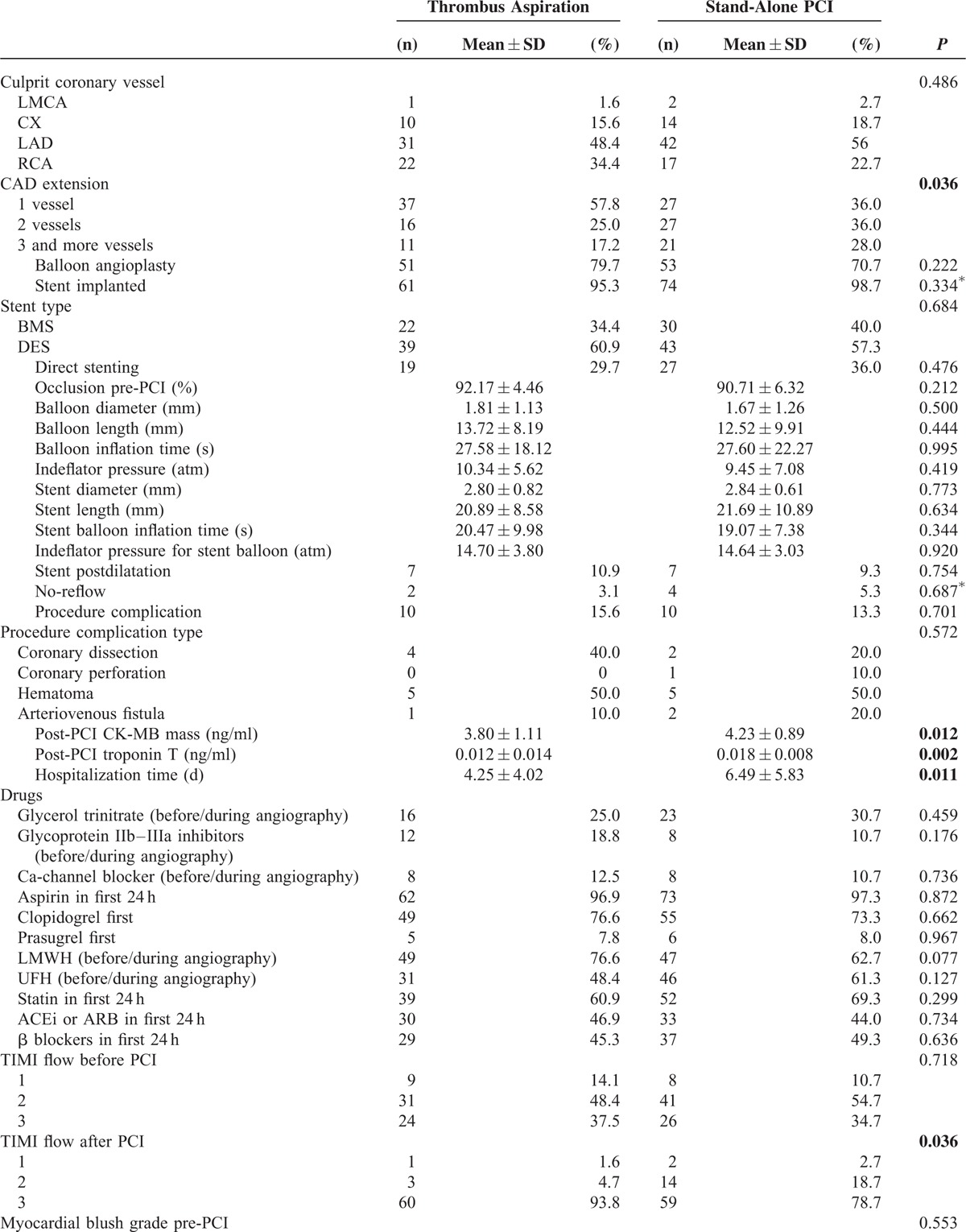
Procedural Characteristics, Angiographic, and Echocardiographic Results of Study Population

**TABLE 2 (Continued):**
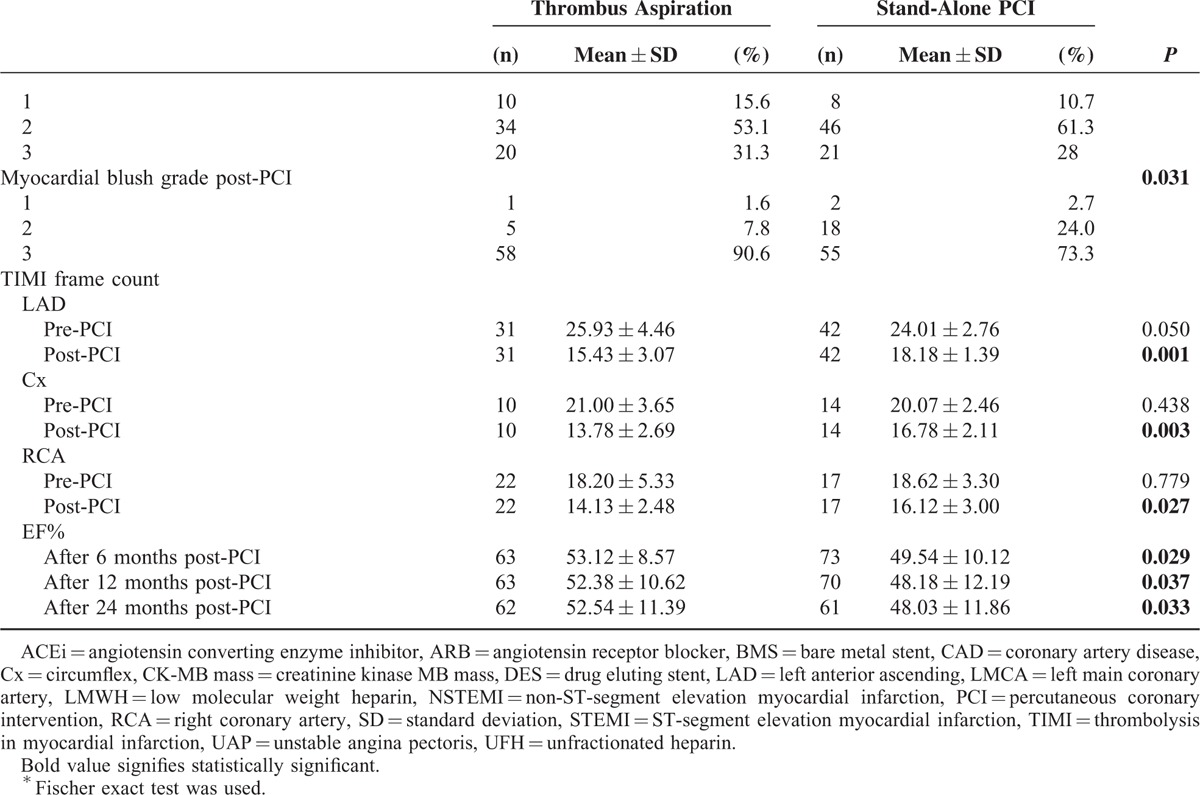
Procedural Characteristics, Angiographic, and Echocardiographic Results of Study Population

**TABLE 3:**
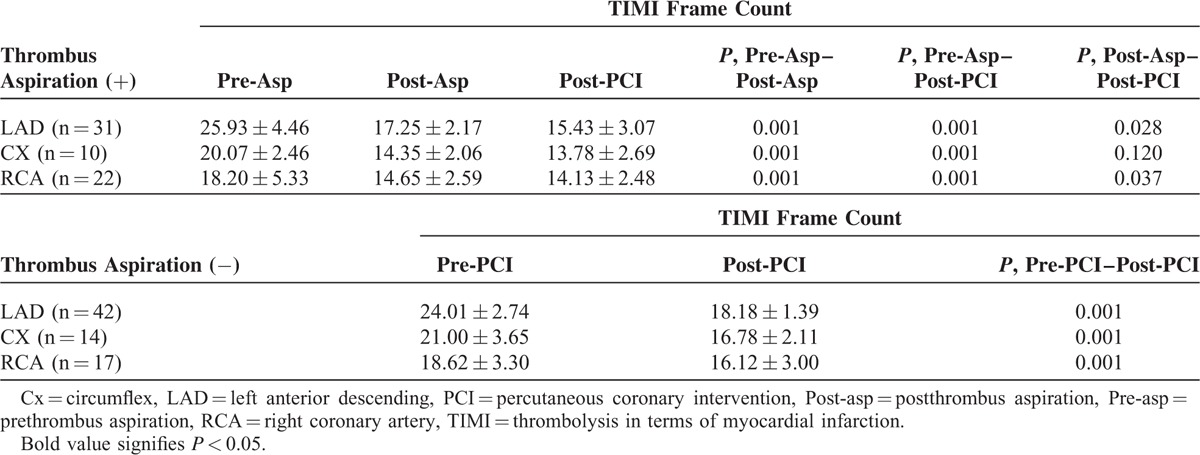
Comparison of TIMI Frame Counts of All Patients

**FIGURE 2:**
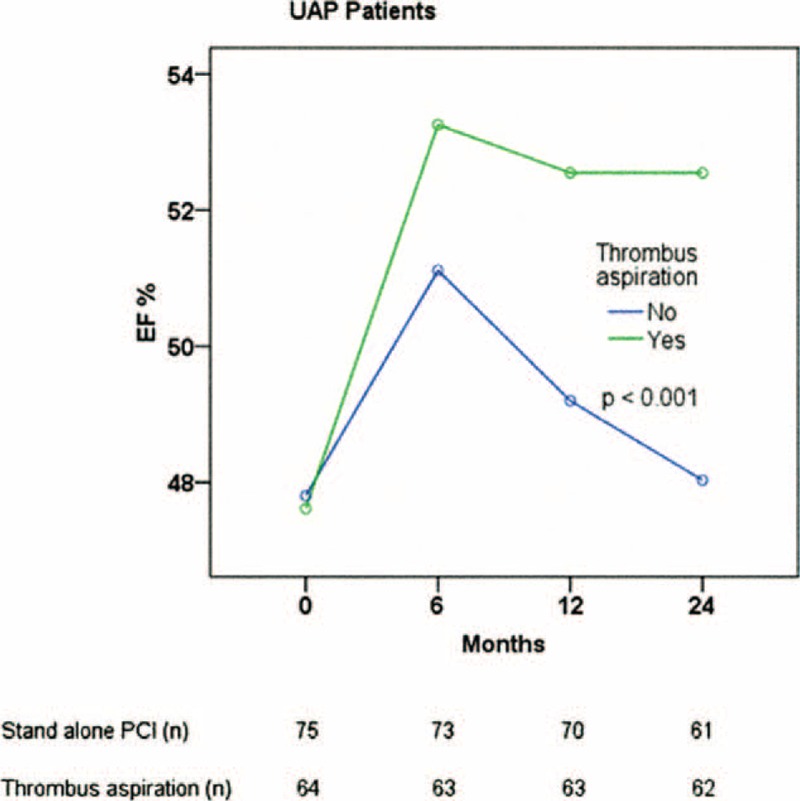
Two-year LVEF according to the use of thrombus aspiration in UAP patients. *P* values come from repeated measured analysis of variance.

At 6, 12, and 24 months post-PCI, the mean LVEF was significantly higher in the group 1 versus the group 2 (Figure 2).

In-hospital mortality, stroke, stent thrombosis, major bleeding, recurrent MI, onset of atrial fibrillation (AF) or ventricular tachycardia/ventricular fibrillation (VT/VF), and acute renal failure were not significantly different between the 2 groups (Table 4).

**TABLE 4:**
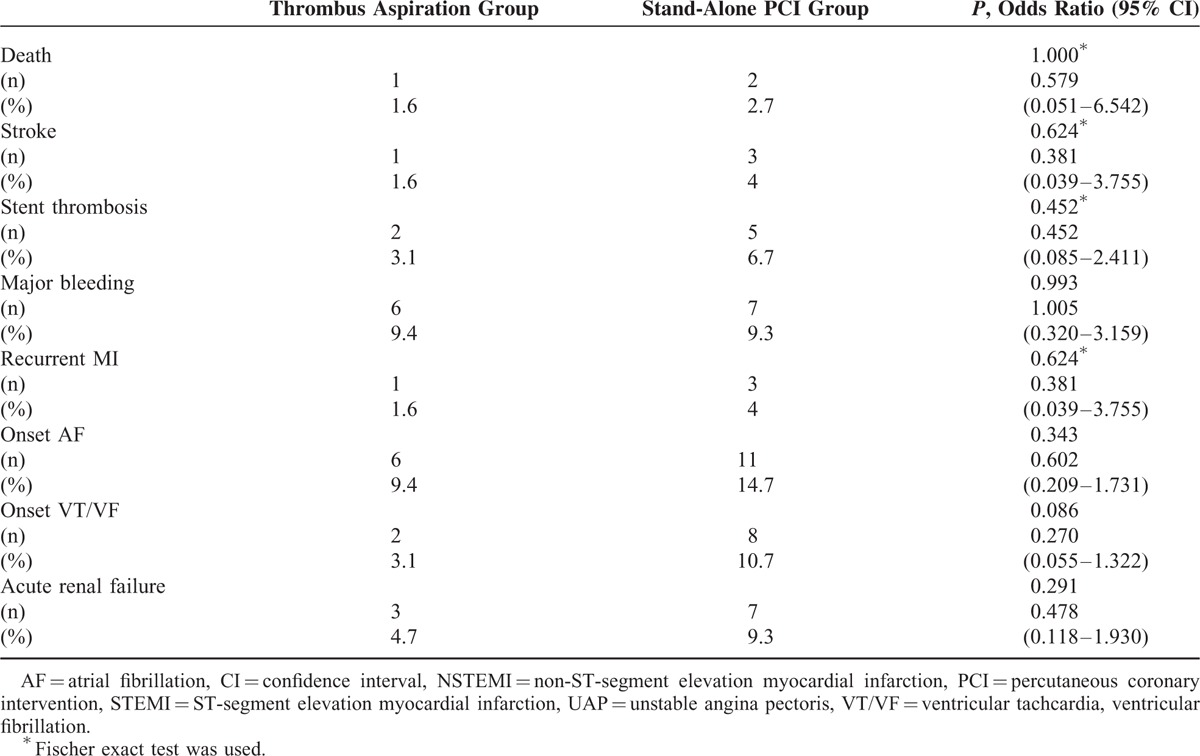
In-Hospital Complications

Death, stroke, bleeding complications, recurrent MI, stent thrombosis, occurrence of AF, occurrence of VT/VF, new renal dialysis, new CABG, rehospitalization for HF, CPM implantation, ICD or CRT-D/P implantation were assessed within 30 months following PCI. Death was significantly lower in the group 1 (unadjusted OR: 0.29, 95% CI: 0.09–0.93, *P* = 0.030). In a multiple logistic regression model adjusted for age, sex, systolic blood pressure, glomerular filtration rate, multivessel CAD, GRACE score at admission, initial TIMI flow, LVEF at 6 months, or concomitant use of GP IIb–IIIa inhibitors, TA was associated with a significant reduction in 30-month mortality (adjusted OR: 7.36, 95% CI: 1.20–45.10, *P* = 0.031). Stroke, recurrent MI, occurrence of AF, occurrence of VT/VF, ICD implantation, rehospitalization for HF, new CABG were also significantly lower in the group 1 than in the group 2 (Table 5).

**TABLE 5:**
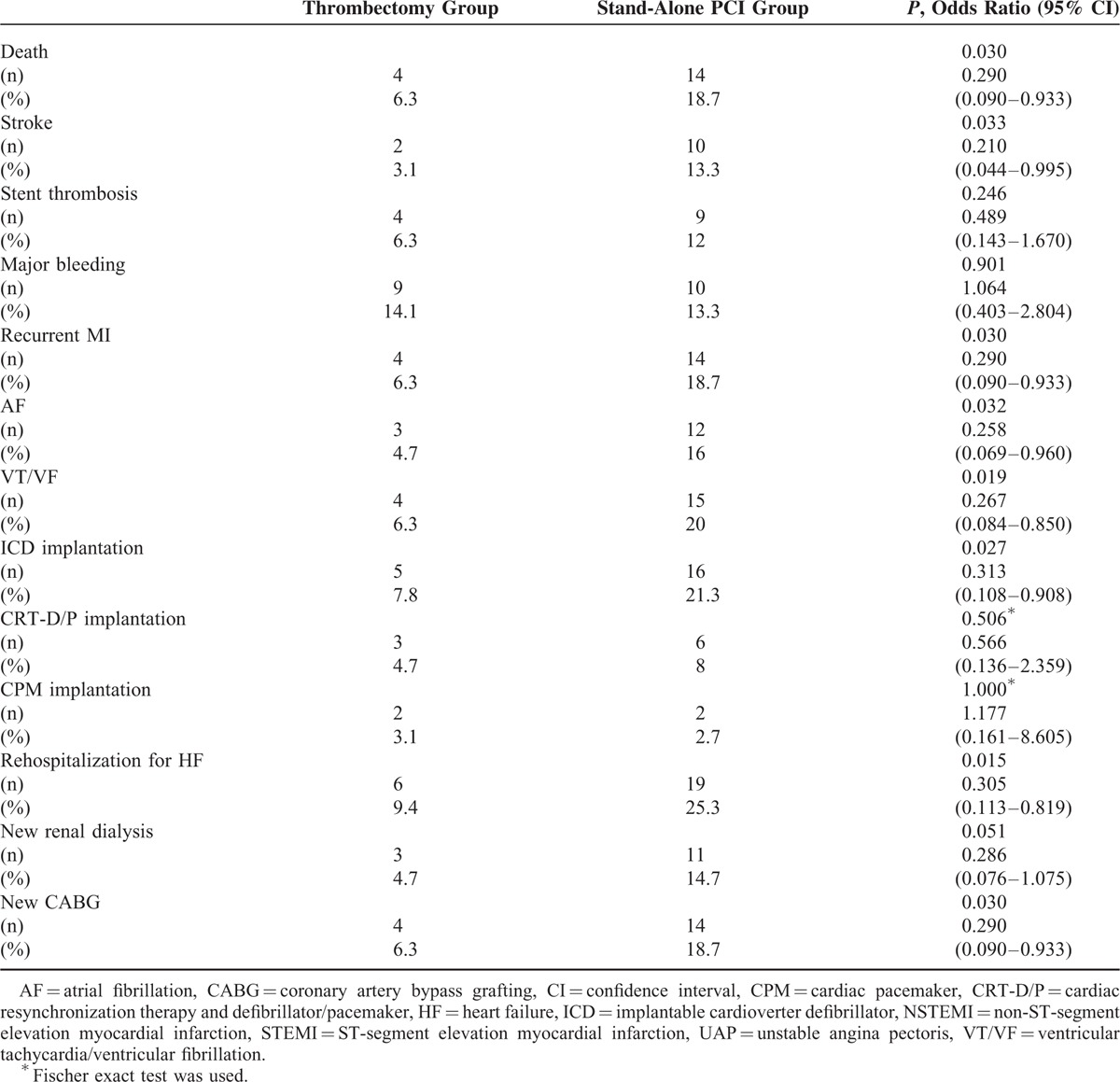
Comparison of Complications Over 30 Months Following in Patients Undergoing Percutaneous Coronary Intervention (PCI)

**FIGURE 3:**
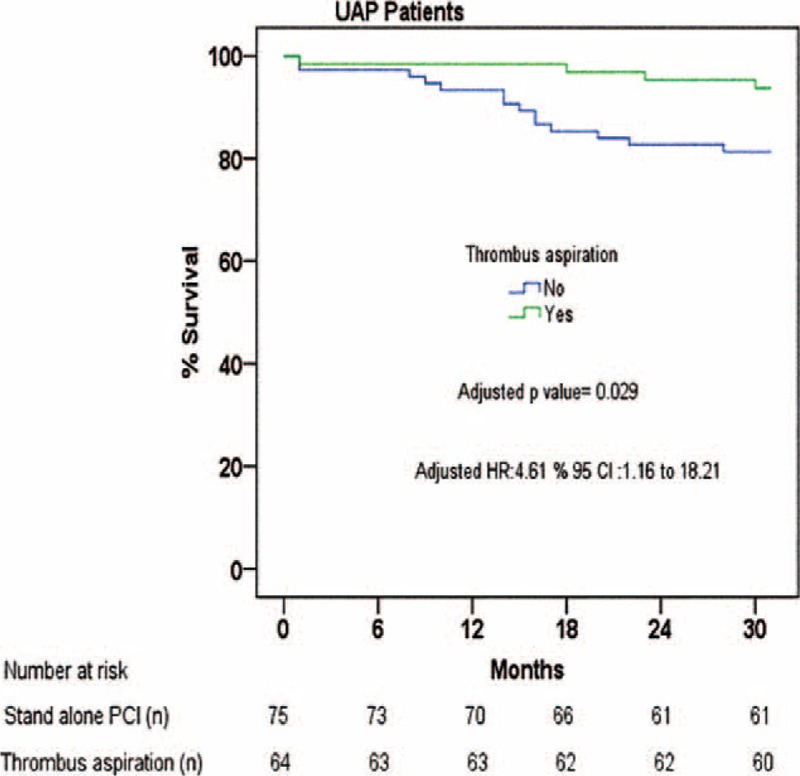
Kaplan–Meier curves for overall survival up to 30-month follow-up according to the use of thrombus aspiration in UAP patients. Log-rank: x^2^: 4.83, *P* < 0.028.

During a mean follow-up period of 28.87 ± 6.28 months (30.18 ± 4.16 months in the group 1 vs 27.76 + 7.49 months in the group 2), 18 patients (12.9%) died. Of these, 4 patients were from the group 1 (6.3%) and 14 from the group 2 (18.7%) (unadjusted HR: 3.24, 95% CI: 1.06–9.58, *P* = 0.038). Using Cox multivariate analysis, TA was associated with significantly less long-term mortality even after the same variables were corrected in all UAP patients (adjusted HR: 4.61, 95% CI: 1.16–18.21, *P* = 0.029). The Kaplan–Meier cumulative survive curve appears in Figure 3.

## DISCUSSION

In this trial, the process of manual TA during PCI in patients with UAP and thrombus-containing lesions was found to be associated with better long-term survival and lower rates of stroke, recurrent MI, arrhythmias (AF, VT/VF), rehospitalization for CHF, and new CABG and ICD implantation within 30 months than in patients treated with PCI only. To the best of our knowledge, this study is the first of PCI-treated UAP patients to have demonstrated an association between TA and reduced mortality.

Previous studies assessing the use of TA in STEMI patients and its outcomes have demonstrated different results.^5,8,17–20^ The Thrombus Aspiration during Percutaneous Coronary Intervention in Acute Myocardial Infarction Study (TAPAS) is the only randomized trial to have demonstrated a significant beneficial effect on mortality: an approximately 50% reduction in 1-year mortality.^8^ Conversely, the Thrombus Aspiration in ST-Elevation Myocardial Infarction in Scandinavia (TASTE) study showed that routine thrombectomy use did not reduce 30-day mortality.^21^ Similar conflicting results were demonstrated in NSTEMI patients. Vlaar et al^10^ found that manual TA was associated with a significant reduction of TIMI thrombus score and an increased rate of TIMI flow grade 3 in NSTEMI patients. Thiele et al^22^ showed that TA in conjunction with PCI in NSTEMI with a thrombus-containing lesion did not precipitate any reduction in microvascular obstruction. Hermens et al^11^ published their initial experiences with manual TA in 14 patients with stable or UAP and recommended that myocardial perfusion might benefit from TA in patients with stable or unstable angina. Based on these data, current American and European guidelines suggest performing manual TA in only STEMI patients with a class IIa level of evidence B recommendation.^23,24^

In this study, manual TA was associated with a significant reduction of TFC and an increase in the rate of TIMI flow 3 and MBG 3 in the manual TA group. Vlaar et al^10^ found an increase in TIMI 3 flow after TA in NSTEMI patients while Burzotta et al^17^ found significant improvement of MBG in patients with successful TA in STEMI patients. In our study, postprocedural levels of CK-MB mass and troponin T were higher in the stand-alone PCI group than in the TA group. These results show that TA reduces thrombus burden and prevents distal embolization in causing microvascular injury. Thereby, increased microvascular flow improves myocardial reperfusion and clinical outcomes.^25,26^

In the present study, we also evaluated LV functions of UAP patients, and baseline LVEFs were similar between the 2 groups. By the time that LVEFs were changed in 6, 12, and 24 months after PCI in both groups. LVEF was significantly higher in the TA group than in the stand-alone group during 24 months. The LVEF increases in both groups at the 1st echo during follow-up, then goes down and plateaus in the thrombectomy group while declining further in the nonthrobectomy group. We may accept that LVEF is acutely depressed or biomarker release (hibernation) and that it improves after revascularization. Decrease in LVEF in the stand-alone PCI group after 6 months may be explained by higher incidence of complications like recurrent MI in this group. Similarly, Liistro et al^27^ showed that manual TA in the context of primary PCI improves myocardial tissue-level perfusion as well as LV functional recovery and remodeling at 6 months. Conversely, in the myocardial contrast echocardiographical substudy of the REMEDIA trial.^28^ TA was associated with no significant reduction in LV remodeling at 6 months. Despite conflicting results, our findings suggest that TA may help to reduce the infarct size and preserve the myocardial function by protecting microvascular obstruction.

According to our study, TA appears to be a valuable approach to improving outcomes in UAP patients with visible thrombus. In this study, improved procedural outcomes with manual TA were observed, though these improvements were not associated with better hospital mortality or in-hospital complication rates. Although there was no significant difference in relationship to hospital complications, duration of hospitalization was significantly briefer in the TA group. Lower long-term complication rates may be attributed to the use of TA during PCI, since TA may help to reduce infarct size, prevent cardiac remodeling, and/or preserve myocardial function by protecting microvascular obstruction. Lower stroke rates up until the 30-month follow-up in our study can be explained by a significant reduction in the onset of AF or VT/VF.

### Study Limitations

In this study, for UAP patients the decision to perform TA was made after careful consideration by experienced interventional cardiologists. Nevertheless, absent the use of intravascular ultrasound or optical coherence tomography, even experienced operators have a limited ability to distinguish intracoronary thrombus formation from calcified lesions. Our data lack information on culprit lesion characteristics (eg, target-vessel tortuosity). In addition, we have not standardized our determination of whether the aspiration catheter crossed the culprit lesion and lacked information concerning the amount of thrombus material removed. Also, the study population remained small.

## CONCLUSIONS

This study demonstrates that manual TA in the context of UAP is associated with a limited elevation in cardiac enzymes during PCI that minimizes microembolization with a significant improvement both of the coronary artery flow and myocardial perfusion, as assessed by the use angiographic TIMI flow grade, TFC, and MBG. The improvement in tissue perfusion is also associated with a significant improvement in regional and global LV function at 24 months. Our findings further support the use of thrombectomy during PCI in UAP patients given its association with better survival over a 30-month follow-up period. Using thrombectomy given the suspicion of thrombus formation in UAP patients affords better results. Nevertheless, further studies with larger sample sizes are needed to evaluate the clinical value of and long-term prognosis following this procedure.
